# Estimating causes of out-of-hospital deaths in China: application of SmartVA methods

**DOI:** 10.1186/s12963-021-00256-1

**Published:** 2021-05-04

**Authors:** Jinlei Qi, Tim Adair, Hafizur R. Chowdhury, Hang Li, Deirdre McLaughlin, Yunning Liu, Jiangmei Liu, Xinying Zeng, Jinling You, Sonja Firth, Renee Sorchik, Peng Yin, Lijun Wang, Maigeng Zhou, Alan D. Lopez

**Affiliations:** 1 National Center for Chronic and Noncommunicable Disease Control and Prevention, Chinese Center for Disease Control and Prevention, 27 Nanwei Road, Xicheng District, Beijing, 100050 China; 2 Melbourne School of Population and Global Health, The University of Melbourne, 207 Bouverie Street, Carlton, Victoria Australia; 3 Institute for Health Metrics and Evaluation, University of Washington, Seattle, USA

**Keywords:** Verbal autopsy, Cause of death, Surveillance, China

## Abstract

**Background:**

Most deaths in China occur at home, making it difficult to collect reliable cause of death (CoD) information. Verbal autopsy (VA) was applied using the SmartVA tool to a sample of home deaths in China to explore its feasibility as a means of improving the quality of CoD data.

**Methods:**

The study was carried out in 22 districts in 9 provinces, located in north-east, central, and western areas of China during 2017 and 2018. Trained interviewers selected suitable respondents in each household to collect information using the Population Health Metrics Research Consortium (PHMRC) shortened and validated electronic VA questionnaire on tablets. The CoD was diagnosed from the interview data using the SmartVA-Analyze 2.0 software (Tariff 2.0).

**Results:**

Non-communicable diseases (NCDs) dominated the leading causes of death in all age groups and for both sexes. After redistribution of undetermined causes, stroke (24%), ischemic heart diseases (IHD) (21%), chronic respiratory diseases (11%), and lung cancer (6%) were the leading causes of death. The cause fractions for level-one cause categories and ranking of specific causes were similar between SmartVA and results from the Global Burden of Disease (GBD) study.

**Conclusion:**

Evidence from this large pilot study suggests that SmartVA is a feasible and plausible tool and could be a valuable tool to improve the quality and standardization of CoD information across China.

**Supplementary Information:**

The online version contains supplementary material available at 10.1186/s12963-021-00256-1.

## Background

Timely and reliable information on mortality and causes of death (CoD) are essential for the development of national health policy [1, 2]. Although it provides a major benefit for death surveillance, CoD data are either unavailable or often of sub-standard quality in many low- and middle-income countries [3, 4].

China has the world’s largest population, and is facing significant health challenges from an increasing burden of non-communicable diseases. A disease surveillance points (DSP) system provides crucial information on mortality conditions in China, covering 340 million people (approximately 24.3% of the country’s population) [5]. The DSP is part of the Chinese Center for Disease Control and Prevention Cause of Death Reporting System (CDRS), which covers 95% of districts and counties in China.

Although the data generated by the DSP system has a high level of completeness, it faces some challenges. For example, 75% of deaths occur at home, and a further 8.6% of deaths lack sufficient medical or other background information to accurately determine the CoD. In addition, 5.5% of deaths are of individuals who had no contact with medical facilities and thus only a limited medical history is available [6–9]. The community physician or CDC staff often has difficulties assigning a correct CoD for these deaths.

Regular review of existing DSP mortality data and periodic re-investigations are critically important to understand and improve the quality of data. However, little or no systematic reviews have been undertaken in this area, particularly pertaining to the quality of CoD data.

Verbal autopsy (VA) is a practical alternative way to measure CoD in populations where the majority of deaths occur without medical attention or proper medical certification. VA is a method for collecting information about the signs and symptoms an individual experienced before death, along with information on previous illnesses and medical care sought before death. Many countries such as Bangladesh, South Africa, Malaysia, Thailand, and Vietnam have used verbal autopsy to improve the quality of underlying CoD, particularly for out-of-hospital deaths [10–17]. In Bangladesh, the research find SmartVA software can be implemented as a cost-effective alternative to Matlab Medical Assistants to routinely collect and analyze verbal autopsy data in a Health and Demographic Surveillance Systems to generate essential population level COD data for planning. In Thailand, Malaysia, and Vietnam, VA implementation results also provide adequate information to enable estimation of cause-specific mortality indicators [18]. The World Health Organization (WHO) has been encouraging wider use of VA as a method to obtain the CoD in low-resource settings [19].

Therefore, one aim of this study was to explore the feasibility and plausibility of a verbal autopsy tool (SmartVA) as a means of investigating and improving the quality of CoD data for a sample of deaths that occurred outside of health facilities in China. A second aim was to assess how closely the CoD estimates from SmartVA align with estimates from the Global Burden of Disease (GBD) study. Results from this study could inform the development of routine verbal autopsy within the CDRS to help enhance the quality of underlying CoD data.

## Methods

### Study setting

This study was undertaken in two phases. Phase I occurred in 2017 and comprised pilot sites in thirteen districts from five provinces—Shandong, Henan, Hubei, Ningxia, Shaaxi—in east, central, and northwest areas of China. While not nationally representative, the pilot sites were chosen with consideration of the diverse cultural, economic, and socio-demographic characteristics of the population [20, 21] (Additional file 1: Appendix I) across these different provinces. To further evaluate the feasibility of VA in other areas of China, phase II, conducted in 2018, focused on northeast, central, and southwest areas, including nine districts within four additional provinces—Sichuan, Anhui, Guizhou, and Heilongjiang. Pilot sites were chosen using the following criteria: (1) Sites with a high proportion of deaths occurring at home and (2) sites with a crude death rate similar to the average crude death rate of the province from which they were chosen. In phase I, three townships or communities were randomly sampled from pilot areas. In phase II, all townships or communities in pilot areas were selected.

All deaths that occurred at home or in the community (i.e., outside a health facility) in the selected pilot site catchment areas and within a designated timeframe were investigated using SmartVA. In order to minimize recall bias, in adults, only deaths that occurred within 1 year of the date of the interview (phase I: from June 1, 2016, to June 30, 2017; phase II: from June 1, 2017, to June 30, 2018) were eligible [22]. As a result of the low level of child and neonatal mortality, child and neonatal deaths within two and a half years from the date of the interview (January 1, 2015, to June 30, 2017) were initially eligible for inclusion in the study. However, due to a high VA refusal rate for child and neonate deaths in phase I, phase II focused solely on adult deaths.

### Data collection instrument

The automated VA method SmartVA was used in this study. SmartVA is comprised of the Population Health Metrics Research Consortium (PHMRC) shortened VA questionnaire, with data collected via the Open Data Kit (ODK) software, and analyzed using the SmartVA-Analyze (Tariff 2.0) software to determine CoD. Responses are gathered via an electronic tablet. The questionnaire consists of a general information module administered for all decedents, and an age-specific module administered based on the age of the decedent. There are three age-specific modules: adolescent and adult deaths (12 years or more), child deaths (29 days to 11 years), and neonate deaths (0 to 28 days). All questionnaires were translated into Chinese prior to use [12, 23].

### Interview procedure

Two to three local CDC staff from each pilot site were trained by National CDC, who had been trained by staff from the University of Melbourne. These trained staff then trained local interviewers. Local interviewers were chosen from township clinics and included village doctors and other local CDC and community staff with a medical background. The training included a brief introduction to SmartVA, counseling skills, ethical issues, confidentiality and sensitivity training, appropriate interpretation of questions, interview technique, and how to input and output data using the tablet.

Interviewers either visited the home of the deceased, or respondents were invited to local CDCs or town clinics to be interviewed. Acceptable respondents were adult household members (age 18 years or above) who preferably cared for the deceased, or were familiar with the illness and its signs and symptoms before death. In the case of a child death, either parent could respond, but preference was given to the mother.

**Fig. 1:**
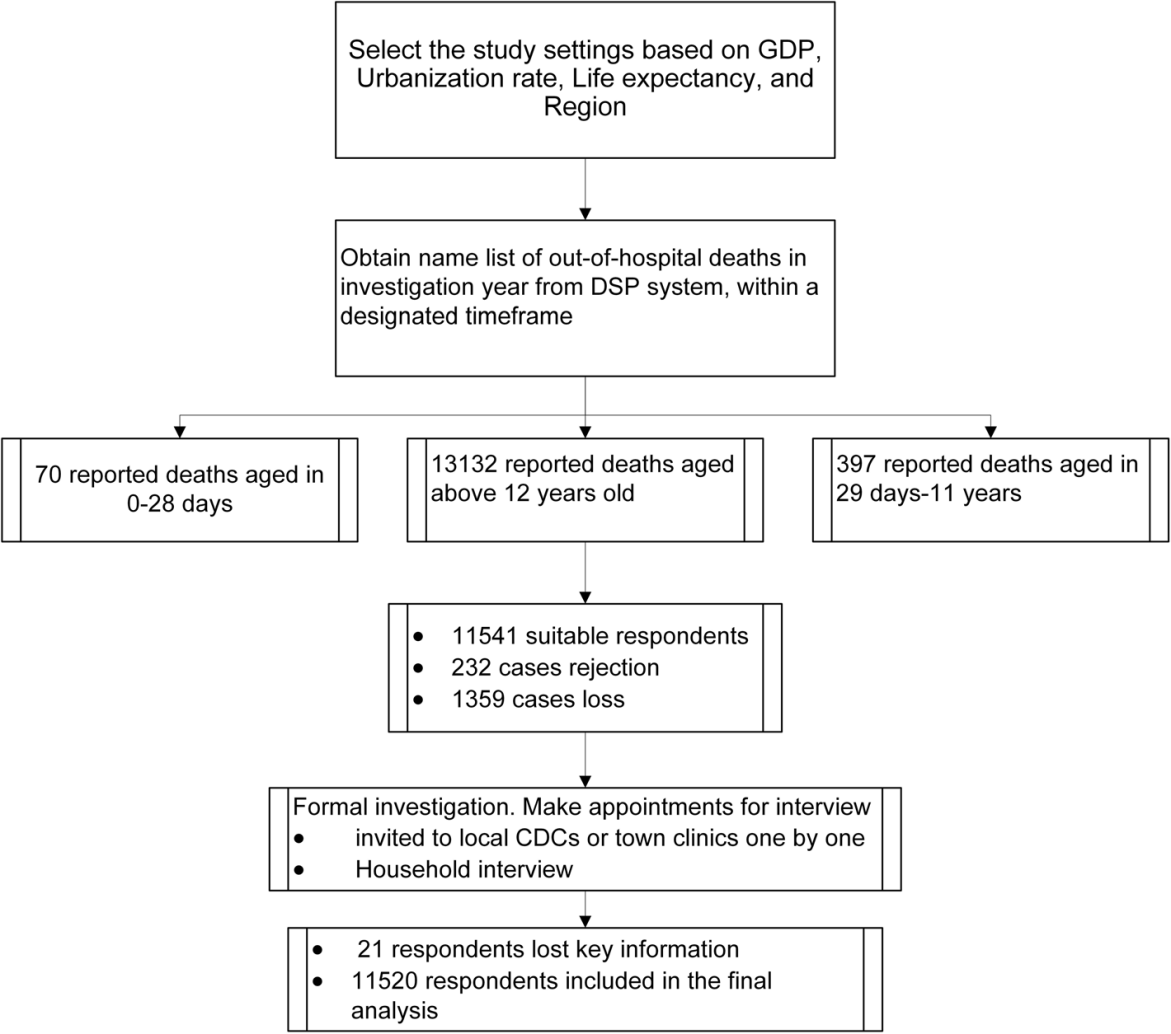
Flowchart for SmartVA study

Quality control was set up at every stage of the study including for survey procedure, data collection and recording, and data analysis. Interviewers and local quality auditors checked interview responses on the same day as the interview. Supervisors from national and provincial CDC offices checked the interviewers’ mastery of interview skills and interviewer effectiveness, and also checked data quality regularly. There was feedback and further re-checking when errors or irregular content was identified during data analysis. Flow chart can be seen in Fig. 1.

### Data management and analysis

The data were exported from ODK Collect using the ODK Briefcase tool. The SmartVA-Analyze 2.0 software was used to assign CoD [24–26], SmartVA-Analyze (Tariff 2.0) has been used in large multi-country validation study and be proved more accurate than other automated diagnostic algorithms, as well as some physicians. SmartVA estimates cause-specific mortality fractions (CSMFs) using algorithms that differ based on whether a population is in a region endemic or non-endemic for malaria and AIDS. The populations were classified as endemic for AIDS but non-endemic for malaria as only 3 indigenous malaria cases were reported in China in 2016, and 7 imported cases were reported in 2017 [27]. CSMFs were calculated from the SmartVA 34-cause list by age group and sex. Deaths assigned “Undetermined” by SmartVA were redistributed to specific causes using an algorithm that is based on GBD estimates. GBD use the redistribution rules to redistribute undetermined cause of death for each age-sex group by proportionate redistribution, statistical models, and expert algorithms and the likelihood of each cause being reported as undetermined [28].

We calculated 95% confidence intervals (CI) to describe the cause-specific mortality fractions from SmartVA estimates, and the formula was as followed:

$$ 95\%\mathrm{confidence}\ \mathrm{intervals}\ \left(\mathrm{CI}\right)=\mathrm{CSMF}\pm 1.96\times \frac{\sqrt{\mathrm{CSMF}\ast \left(\mathrm{CSMF}\right)}}{\mathrm{deaths}} $$

We also present uncertainty intervals (UI) of the cause-specific mortality fractions from GBD estimates [28]. The redistribution of undetermined cases was only performed for all adult ages collectively; CSMFs for specific age groups retain the undetermined cause category.

The results were assessed for plausibility by comparing them to GBD estimates both for all of China and for the nine provinces where the pilot sites were located (Chinese estimates from the Global Burden of Diseases, Injuries, and Risk Factors Study 2017) [29]. Estimates published in the GBD cover all deaths, while the results of this study are specific to home and community deaths. To allow for comparison between the two sources, GBD cause categories (level 3) were mapped to the VA 34-cause list produced by SmartVA. The age and sex distribution of VA deaths in the pilot sites was also compared to that of the DSP and the GBD for the 9 provinces to assess the representativeness of the results.

## Results

### Study population characteristics

As shown in Additional file 1: Appendix II, 13,599 deaths were reported and investigated using VA at the 22 pilot sites. Of these, 2.0% of all respondents refused to participate in the investigation and 1421 (10.4%) deaths were lost to follow up (1359 for adults, 47 for children, 15 for neonates). The main causes of loss to follow up were household members were at work or visiting relatives during the period of investigation; appropriate respondents could not be found; the family moved to another place.

A total of 11,541 adolescent and adult deaths (aged 12 years and above) were followed up with a VA between 2017 and 2018. VAs were excluded if respondents completed less than 95% of the key questions or did not report the age or sex of the deceased (*n*=21). In total, 11,520 (99.8%) VAs for adolescent and adult deaths were analyzed, of which 6359 (55.2%) were male and 5161 (44.8%) were female.

The median age at death was 76 years (inter-quartile range 66-83). The age and sex distribution of deaths from the 9 pilot sites were compared to estimates from the DSP and the GBD for 9 provinces (Table 1). The age distribution of deaths by sex was similar among all three sources, suggesting that the age pattern of deaths in the 9 pilot sites was similar to the mortality pattern seen in all of China. The pilot sites had a slightly larger proportion of deaths concentrated in persons aged 70 and above (and thus a lesser proportion of deaths to those under age 70) compared to GBD 2017 estimates. The distribution of respondents from 9 pilot sites and DSP deaths by sex, age groups, and urban-rural (Additional file 1: Appendix III) suggest that the urban-rural pattern of deaths in the 9 pilot sites was similar to their DSP.

**Table 1:** Distribution of adult deaths from SmartVA, compared to DSP and China GBD 2017, by sex and age groups (%)

Age groups	Both			Male			Female		
	SmartVA	DSP	GBD	SmartVA	DSP	GBD	SmartVA	DSP	GBD
12-14	0.1	0.1	0.1	0.2	0.1	0.2	0.1	0.1	0.1
15-19	0.1	0.2	0.3	0.2	0.3	0.3	0.1	0.2	0.2
20-24	0.3	0.3	0.3	0.4	0.4	0.4	0.1	0.2	0.2
25-29	0.5	0.6	0.8	0.6	0.7	1.0	0.5	0.4	0.6
30-34	0.4	0.7	1.0	0.6	0.9	1.2	0.3	0.5	0.7
35-39	0.7	1.0	1.0	0.8	1.3	1.2	0.7	0.7	0.7
40-44	1.4	1.8	1.7	1.7	2.2	2.1	1.0	1.2	1.2
45-49	2.7	3.4	3.5	3.3	4.1	4.2	2.0	2.4	2.5
50-54	4.2	5.0	5.3	5.3	6.0	6.2	2.9	3.6	4.0
55-59	4.1	4.7	4.4	5.3	5.7	5.1	2.8	3.4	3.3
60-64	8.0	8.5	9.2	9.2	9.9	10.6	6.5	6.5	7.4
65-69	10.4	10.2	12.0	11.7	11.4	13.2	8.8	8.5	10.4
70-74	12.7	11.5	11.8	13.3	12.2	12.4	12.1	10.5	10.9
75-79	16.3	14.3	13.9	16.3	14.3	13.9	16.4	14.3	13.9
80-84	18.0	16.6	16.0	16.2	14.9	14.5	20.2	19.0	18.1
85+	19.9	21.0	18.7	15.2	15.6	13.5	25.7	28.5	25.9
Total	11,520	1,764,929	10,249,326	6,359	1,024,158	5,931,469	5161	740,771	4,317,857
	(100.0)	(100.0)	(100.0)	(100.0)	(100.0)	(100.0)	(100.0)	(100.0)	(100.0)

### Causes before and after redistribution of undetermined causes

Additional file 1: Appendix IV shows the distribution of SmartVA respondents, determined and undetermined cause of death respondents by sex and age groups. It can be seen from the comparison that although the difference in other age groups is not obvious, the proportion of undetermined cause is much higher than the determined causes in above 85 years old, especially in female.

Table 2 shows the CSMFs for all home deaths for adults estimated by SmartVA. Stroke, ischemic heart disease (IHD), and chronic respiratory disease were the three leading causes of death in both men and women before and after redistribution, accounting for more than half of all adult deaths. Lung cancer was the fourth ranked cause for men (8.1%) while only the tenth-ranked cause for women (2.2%), whose fourth-ranked cause was diabetes (3.6%). The proportion of undetermined causes for women (11.9%) was slightly higher than for men (8.3%).

**Table 2:** Adult cause-specific mortality fractions before and after redistribution of undetermined cause category by sex, 2017-2018 (%)

Cause	Both		Male		Female	
	Before	After	Before	After	Before	After
Stroke	23.1	24.4	21.1	22.0	25.6	27.0
Ischemic heart disease	20.1	21.2	17.9	18.6	22.9	24.1
Chronic respiratory	9.7	10.6	10.5	11.1	8.8	9.7
Lung cancer	5.2	5.5	7.8	8.1	1.9	2.2
Other non-communicable diseases	3.1	3.8	3.7	4.2	2.3	3.2
Pneumonia	2.8	3.3	3.3	3.7	2.2	2.8
Prostate cancer	2.9	3.0	5.2	5.3	0.0	0.0
Diabetes	2.6	2.9	2.0	2.2	3.3	3.6
Leukemia/lymphomas	2.3	2.5	2.2	2.4	2.4	2.6
Other injuries	2.1	2.5	2.0	2.2	2.3	2.7
Cirrhosis	2.1	2.4	2.9	3.1	1.2	1.5
Esophageal cancer	2.0	2.1	2.3	2.5	1.6	1.7
Falls	2.0	2.1	2.0	2.1	1.9	2.1
Stomach cancer	1.3	1.5	1.3	1.4	1.4	1.6
Chronic kidney disease	1.2	1.5	1.3	1.5	1.1	1.5
Cervical cancer	1.3	1.4	0.0	0.0.	3.0	3.0
Road traffic	1.1	1.3	1.4	1.5	0.8	0.9
Poisonings	0.9	1.2	1.0	1.3	0.8	1.1
Tuberculosis	0.9	1.1	1.1	1.3	0.6	0.8
Other cancers	0.6	1.0	0.6	0.9	0.7	1.1
Diarrhea/dysentery	0.5	1.0.	0.5	0.9	0.6	1.2
Breast cancer	0.5	0.6	0.0	0.0	1.1	1.2
Suicide	0.5	0.6	0.6	0.7	0.5	0.6
Drowning	0.4	0.6	0.5	0.6	0.3	0.5
Other infectious diseases	0.3	0.5	0.2	0.4	0.4	0.7
Other cardiovascular diseases	0.0	0.4	0.0	0.3	0.0	0.5
Fires	0.2	0.3	0.2	0.3	0.2	0.3
AIDS	0.1	0.3	0.1	0.3	0.1	0.3
Homicide	0.1	0.3	0.1	0.2	0.1	0.3
Colorectal cancer	0.0	0.3	0.0	0.3	0.0	0.4
Maternal	0.0	0.0	0.0	0.0	0.1	0.1
Undetermined	9.9	0.0	8.3	0.0	11.9	0..0.

### Causes (%) by age and sex

Table 3 shows the top five leading causes of death, along with the proportion of undetermined deaths, for five broad age groups by sex. Deaths due to undetermined causes could only be redistributed for all adult deaths collectively, and not for each broad age group. The two leading causes of death among 12 to 49 year old women were cervical cancer (8.5%) and stroke (8.1%). Cervical cancer was the fifth leading CoD among women aged 50 to 59 (5.2%) and the third leading CoD among women aged 60 to 69 (5.2%). Stroke was the leading cause and IHD the second leading CoD in all age-groups for men, and for all women aged 50 and above. Lung cancer was the third leading CoD among men aged 50 to 59 years (14.6%) and 60 to 69 years (15.0%), and the fifth leading cause among men aged 70-79 years (6.1%). Cirrhosis (7.6%), falls (6.4%), and road traffic injury (6.2%) were some of the leading causes of death among men at younger ages (12-49 years). Cirrhosis was the fourth-ranked CoD among men aged 50 to 59 years (5.8%). Additional file 1: Appendix V shows percentage of the top five leading causes of death, and their confidence interval (CI) for SmartVA, and percentage of the top five leading causes of death, and their uncertainty interval (UI) for GBD results by GBD specific age groups.

**Table 3:** Percent of adult deaths due to the five leading causes of death and undetermined causes

Gender	Ranking	12-49 years			50-59 years			60-69 years			70-79 years			80+ years		
		CoD	% of total	GBD for 9 provinces	CoD	% of total	GBD for 9 provinces	CoD	% of total	GBD for 9 provinces	CoD	% of total	GBD for 9 provinces	CoD	% of total	GBD for 9 provinces
Male	1	Stroke	9.1	11.3	Stroke	15.3	17.6	Stroke	20.5	21.4	Stroke	23.6	24.4	Stroke	23.8	23.4
	2	IHD	8.7	10.6	IHD	14.6	13.5	IHD	15.9	13.9	IHD	19.2	15.8	IHD	21.2	22.8
	3	Cirrhosis	7.6	3.5	Lung cancer	14.6	10.3	Lung cancer	15.0	12.0	Chronic respiratory	12.4	12.5	Chronic respiratory	15.2	15.2
	4	Falls	6.4	3.5	Cirrhosis	5.8	3.5	Chronic respiratory	7.1	7.1	Prostate cancer	6.4	1.1	Pneumonia	4.5	2.1
	5	Road traffic	6.2	16.5	Chronic respiratory	5.7	3.6	Prostate cancer	6.3	0.6	Lung cancer	6.1	9.1	Other non-communicable diseases	4.1	9.4
	Undetermined	Undetermined	10.5	0.0	Undetermined	6.1	0.0	Undetermined	5.9	0.0	Undetermined	6.9	0.0	Undetermined	11.8	0.0
	Total	Total	485	236,445	Total	673	262,201	Total	1,324	592,448	Total	1,879	660,879	Total	1,998	636,735
Female	1	Cervical cancer	8.5	3.6	Stroke	20.7	18.0	Stroke	21.7	22.0	Stroke	29.7	24.7	Stroke	26.9	22.5
	2	Stroke	8.1	10.4	IHD	17.6	11.6	IHD	21.4	15.4	IHD	23.9	19.1	IHD	25.1	26.5
	3	IHD	8.1	8.0	Leukemia/lymphomas	7.2	3.5	Cervical cancer	5.2	1.5	Chronic respiratory	8.6	10.7	Chronic respiratory	11.6	13.2
	4	Leukemia/lymphomas	7.7	2.6	Diabetes	5.2	1.9	Leukemia/lymphomas	5.0	0.7	Diabetes	3.5	2.1	Other injuries	3.1	0.2
	5	Breast cancer	7.3	5.6	Cervical cancer	5.2	3.5	Chronic respiratory	4.8	6.4	Pneumonia	2.5	1.0	Diabetes	2.7	1.0
	Undetermined	Undetermined	12.1	0.0	Undetermined	8.6	0.0	Undetermined	9.9	0.0	Undetermined	9.0	0.0	Undetermined	14.8	0.0
	Total	Total	246	103,906	Total	290	127,584	Total	787	334,850	Total	1471	455,514	Total	2367	733,885

The proportion of deaths with an undetermined cause was higher among women compared to men, and was highest in the youngest and oldest age groupings. In women, the range spanned from 8.6% among women aged 50 to 59 to 14.8% among women aged 80 years and above. In men, the proportion of deaths of undetermined cause ranged from 5.9% of deaths in men aged 60 to 69 compared to 11.8% of deaths in men aged 80 years and above.

### Comparison with global burden of disease

As shown in Table 4, the two leading causes of death in the pilot areas, stroke and IHD, are also the leading causes of death and constitute similar proportions of deaths in the GBD for China as a whole, and for the GBD for the nine provinces that contained the pilot sites. The proportion of deaths attributed to these causes was slightly higher for the pilot areas compared to the two GBD estimates (stroke: 20.6% GBD, 21.6% GBD for 9 provinces, 24.4% SmartVA; IHD: 21.2% SmartVA, 18.0% GBD for 9 provinces, 17.1% GBD).

**Table 4:** Distribution of SmartVA causes for adult deaths compared to China GBD 2017 by sex, 2017-2018

CoD	Both			Male			Female		
	SmartVA	GBD for 9 provinces	GBD for China	SmartVA	GBD for 9 provinces	GBD for China	SmartVA	GBD for 9 provinces	GBD for China
Stroke	24.4	21.6	20.6	22	21.4	20.3	27	21.9	20.9
Ischemic Heart Disease	21.2	18	17.1	18.6	16.4	15.9	24.1	20.2	18.7
Chronic Respiratory	10.6	9.8	9.8	11.1	9.8	9.8	9.7	9.9	9.9
Other Cancers	1	8.6	8.9	0.9	9.6	10	1.1	7.2	7.4
Other Non-communicable Diseases	3.8	7.3	8.1	4.2	5.8	6.4	3.2	9.3	10.4
Lung Cancer	5.5	7.1	6.8	8.1	8.3	8	2.2	5.3	5
Other Cardiovascular Diseases	0.4	4.4	5	0.3	3.8	4.3	0.5	5.2	6
Stomach Cancer	1.5	3.8	3.5	1.4	4.5	4.1	1.6	2.8	2.6
Road Traffic	1.3	2.5	2.5	1.5	3.3	3.3	0.9	1.5	1.5
Esophegal cancer	2.1	2.4	2.1	2.5	3.1	2.6	1.7	1.5	1.3
Colorectal Cancer	0.3	1.6	1.8	0.3	1.7	1.9	0.4	1.6	1.8
Chronic kidney disease	1.5	1.5	1.7	1.5	1.4	1.6	1.5	1.6	1.9
Cirrhosis	2.4	1.4	1.5	3.1	1.7	1.8	1.5	1	1
Diabetes	2.9	1.3	1.5	2.2	1.1	1.2	3.6	1.7	1.8
Pneumonia	3.3	1.2	1.5	3.7	1.2	1.5	2.8	1.3	1.6
Falls	2.1	1.1	1.3	2.1	1.2	1.3	2.1	0.9	1.3
Suicide	0.6	1.5	1.3	0.7	1.5	1.3	0.6	1.5	1.2
Breast Cancer	0.6	0.8	0.9	0	0	0	1.2	1.9	2
Other Injuries	2.5	0.7	0.7	2.2	0.9	0.9	2.7	0.3	0.4
Drowning	0.6	0.5	0.5	0.6	0.5	0.5	0.5	0.4	0.4
Leukemia/Lymphomas	2.5	0.5	0.5	2.4	0.5	0.5	2.6	0.5	0.5
Prostate Cancer	3	0.5	0.5	5.3	0.8	0.9	0	0	0
Cervical Cancer	1.4	0.4	0.4	0	0	0	3	1	1
Other Infectious Diseases	0.5	0.3	0.4	0.4	0.3	0.4	0.7	0.3	0.3
Tuberculosis	1.1	0.3	0.4	1.3	0.4	0.5	0.8	0.2	0.3
AIDS	0.3	0.3	0.3	0.3	0.4	0.4	0.3	0.2	0.2
Poisonings	1.2	0.3	0.2	1.3	0.3	0.2	1.1	0.3	0.2
Fires	0.3	0.1	0.1	0.3	0.1	0.1	0.3	0.1	0.1
Homicide	0.3	0.1	0.1	0.2	0.1	0.2	0.3	0.1	0.1
Bite of Venomous Animal	0	0	0	0	0	0	0	0	0
Diarrhea/Dysentery	1	0	0	0.9	0	0	1.2	0	0
Maternal	0	0	0	0	0	0	0.1	0	0.1

**Fig. 2:**
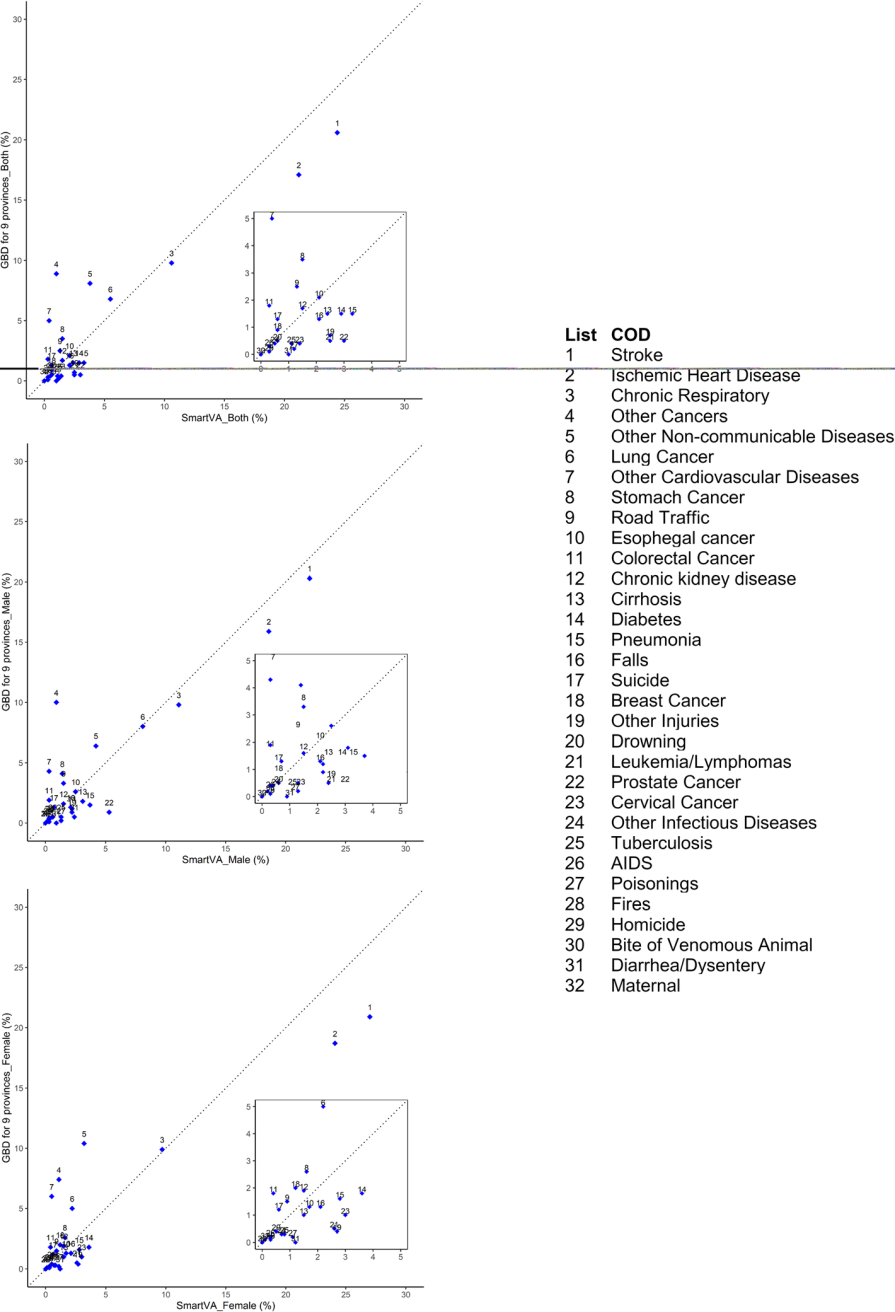
Cause-specific mortality fraction (CSMF) scatter plots with both sex (**a**) and male (**b**) and (**c**) in 2017-2018

Differences between GBD and SmartVA were observed for the rank/fraction of other cancers, other non-communicable diseases, and other cardiovascular diseases (Fig. 2). These causes accounted for a higher proportion of deaths in China GBD for nine provinces (8.6%, 7.3%, and 4.4% respectively) compared to SmartVA pilot areas (1.0%, 3.8%, 0.4% respectively). Liver cancer (5.0%), pancreatic cancer (1.0%), and brain and nervous system cancer (1.0%) were the leading “other cancers” in the GBD for nine provinces for men, and liver cancer (3.0%), pancreatic cancer (1.0%), brain and nervous system cancer (1.0%), and ovarian cancer (1.0%) were leading GBD cancers for women; none of these cancers are a specific cause in the SmartVA cause list, which explains a large portion of the discrepancy between the two sources.

Differences between the SmartVA sites and the GBD for 9 provinces are greatest in the younger age groups (12 to 49 years) (Table 3). The leading CoD for men aged 12 to 49 years was road traffic accidents (16.5%) for GBD in the 9 provinces compared with stroke (9.1%) for SmartVA. Stroke (11.3%) and IHD (10.6%) were the second and third leading causes of death in men in this age group for GBD for 9 provinces, compared with the first (9.1%) and second (8.7%) leading cause for SmartVA. Stroke (10.4%) was the leading cause younger women (12 to 49 years) for GBD in the 9 provinces compared to cervical cancer (8.5%) for SmartVA. IHD (8.0%) and breast cancer (5.6%) were the second and third leading causes of death in GBD for 9 provinces compared to stroke (8.1%) and IHD (8.1%) in SmartVA. Other causes of death, excluding prostate cancer and leukemia/lymphomas, for different age groups are similar in rank between the sources.

According to the SmartVA results, 6.2% of deaths were due to communicable and neonatal, maternal, and nutritional diseases (GBD group 1); 84.9% of deaths due to non-communicable disease (NCDs) (GBD group 2); and 8.9% of deaths due to injuries (GBD group 3). SmartVA attributed a lower proportion of deaths to NCDs compared with the GBD 2017 China estimates (group 2: 89.5%) and GBD estimates for 9 provinces (group 2: 91.1%) (Table 5).

**Table 5:** Comparison of adult causes of death between SmartVA and GBD 2017

GBD	GBD for China (%)	GBD for 9 provinces	SmartVA (%)
Communicable and neonate, maternal, and nutritional diseases	3.5	2.2	6.2
Non-communicable disease	89.5	91.1	84.9
Injuries	7	6.8	8.9

## Discussion

This pilot study was conducted to determine whether SmartVA could be an appropriate and effective tool for VA in China in terms of training and implementation and obtaining reliable CoD data. The study also aimed to provide an empirical assessment of the GBD estimation methods used in China.

Non-communicable diseases (NCDs) dominate the leading causes of death in all age groups and for both sexes, which highlights the importance of focusing management and prevention measures on NCDs [28]. Stroke, ischemic heart disease, and chronic respiratory diseases were the top three CoDs in the majority of age groups in men and women. The predominance of stroke and IHD as the leading causes across most adult age groups indicates possible risk factors (such as hypertension and tobacco use) [21], and addressing these in early adulthood needs to be prioritized [29]. Lung cancer and chronic respiratory diseases featured in the top five leading causes of death in males aged between 50 and 79 years, and cirrhosis in ages 50-59 years, which suggests that a high prevalence of tobacco and alcohol use is likely in the study areas [28, 30]. Cervical cancer was the leading cause of death among young women aged 12 to 49 years. Screening for cervical cancer and provision of the HPV vaccine for young women should be prioritized to reduce the burden of this disease [31]. Targeting strategies to reduce risky behavior in men at younger ages (12-49 years) is also needed to address falls and road traffic injury deaths in this age group.

Our study shows that the distributions of the three broad cause categories from SmartVA are quite similar and comparable with the GBD 2017 estimates [26], with the most notable difference being a higher proportion of deaths from communicable diseases in the pilot sites. For more specific causes of death, there are also quite similar cause proportions in this study and the GBD, with the exception of residual cause categories (e.g., other non-communicable, other cardiovascular, other cancers), and for causes in specific ages by sex (e.g., cirrhosis). Differences are most likely because (1) the GBD estimates CSMFs for all deaths in the 9 provinces or all of China, while SmartVA estimates them for home deaths in the pilot sites only—deaths of younger people and some specific causes such as road traffic accidents are more likely to occur at hospital or on the way to hospital rather than at home [32], and home deaths are not representative of all deaths. (2) Some related causes of death are difficult to distinguish with VA, for example, cirrhosis and liver cancer. The higher proportions of deaths in SmartVA due to group 1 causes may be the result of misclassification of pneumonia and chronic respiratory deaths. (3) Possible errors in GBD estimates because they are largely model-driven and lack reliable cause of death data as an input (which is partly why verbal autopsy is being piloted in this study).

The proportion of undetermined CoD increased with age. Increased number of co-morbidities at older ages makes ascertaining the CoD relatively more difficult. Although it is difficult to eliminate undetermined causes, they can be reduced through close monitoring and evaluation. The proportion of deaths with an undetermined cause, a good measure of the quality of VA data collected, was only 10%, which is at the lower end of the range of 10-20% that is commonly found [33]. This shows that a high-quality training and interview process along with close supervision and monitoring were maintained.

Overall, in this pilot study, the average unit price of each interview is about $8 US dollars, and the average time of each interview is 10-15 min. The results from this study show that the VA findings are plausible and do not provide any significantly abnormal results. Furthermore, the age distribution of adult deaths is similar between Smart VA pilot sites, DSP reports, and GBD estimation. Moreover, if it is feasible in broader areas, Smart VA interview can be potentially embedded in the national death cause surveillance system in the future, to facilitate the determination of cause of death at home.

### Strength and challenges

There are several strengths to this study. The study included a large number of adult home deaths. The refusal and loss to follow-up rates were minimal. The distributions of age groups and sex were similar to and comparable with those of the national population. The VA interviews were conducted by well-trained field staff using a validated electronic questionnaire, and analysis was carried out by automated computer software—SmartVA Analyze 2.0—to assign CoD.

Challenges were also encountered during the rolling out period of this pilot study. The interviews were a heavy workload for health staff, who were already busy with routine work. VA interviews were often conducted over weekends or in the evenings. Therefore, it was sometimes difficult to obtain appointments with the correct or best respondent to ensure good quality interviews. In addition, pilot areas were not selected to be nationally representative. However, the age distribution from this study is similar to that of DSPs in China.

## Conclusion

We have systematically described applying SmartVA in China. Given the large and rising number of deaths being reported, the identification of the causes of death is becoming increasingly important. For out-of-facility deaths, verbal autopsy is a practical and feasible way to achieve this. Our study shows that good interviewer training and supervision, the use of convenient electronic tools to collect information (tablets) and the automated SmartVA analysis tool [34], can lead to a low undetermined cause proportion (10%) and provide a stable and reliable result. The study also indicates the potential for this method to be applied more widely throughout China in order to make meaningful and sustained reductions in the proportion of unusable causes of death to better aid policy making [35, 36].

## Supplementary Information

**Additional file 1: Appendix I.** Gross Regional Product (GRP), urbanization rate, and life expectancy of the 9 pilot provinces. **Appendix II.** SmartVA Interview results from 9 pilot Provinces, 22 Districts between 2017 and 2018. **Appendix III.** The distribution of adult deaths from SmartVA and DSP, by sex ,age groups and urben-rural (%). **Appendix IV.** The distribution of adult deaths from determined and undetermined cause of death by sex and age groups (%). **Appendix V.** Percent of adult deaths due to the five leading causes of death and undetermined causes between SmartVA and GBD for China.

## Data Availability

The data supporting the findings of this study are available from contact of the corresponding author.

## Abbreviations

SmartVA: Smart verbal autopsy
CoD: Cause of death
PHMRC: Population Health Metrics Research Consortium
NCDs: Non-communicable diseases
IHD: Ischemic heart diseases
GBD: Global Burden of Disease
DSPs: Disease surveillance points system
CDRS: Chinese Center for Disease Control and Prevention Cause of Death Reporting System
WHO: World Health Organization
CSMFs: Cause-specific mortality fractions
CDC: Centers for Disease Control and Prevention
ODK: Open Data Kit software

## References

[1] AbouZahr C, de Savigny D, Mikkelsen L, Setel PW, Lozano R, Lopez AD (2015). Towards universal civil registration and vital statistics systems: the time is now. Lancet.

[2] WHO (2013). Strengthening civil registration and vital statistics for births, deaths and causes of death: resource kit.

[3] Mahapatra P, Shibuya K, Lopez AD, Coullare F, Notzon FC, Rao C, Szreter S (2007). Civil registration systems and vital statistics: successes and missed opportunities. Lancet.

[4] Mikkelsen L, Phillips DE, AbouZahr C, Setel PW, de Savigny D, Lozano R, Lopez AD (2015). A global assessment of civil registration and vital statistics systems: monitoring data quality and progress. Lancet.

[5] Liu S, Wu X, Lopez AD, Wang L, Cai Y, Page A, Yin P, Liu Y, Li Y, Liu J, You J, Zhou M (2016). An integrated national mortality surveillance system for death registration and mortality surveillance, China. Bull World Health Organ.

[6] Noncommunicable Disease Control and Prevention, Chinese Center for Disease Control and Prevention & Center for Health Statistics and Information, National Health and Family Planning Commission (2015). Chinese Mortality Surveillance Dataset 2014[M].

[7] Noncommunicable Disease Control and Prevention, Chinese Center for Disease Control and Prevention & Center for Health Statistics and Information, National Health and Family Planning Commission (2016). Chinese Mortality Surveillance Dataset 2015[M].

[8] Noncommunicable Disease Control and Prevention, Chinese Center for Disease Control and Prevention & Center for Health Statistics and Information, National Health and Family Planning Commission (2017). Chinese Mortality Surveillance Dataset 2016[M].

[9] Noncommunicable Disease Control and Prevention, Chinese Center for Disease Control and Prevention & Center for Health Statistics and Information, National Health and Family Planning Commission (2018). Chinese Mortality Surveillance Dataset 2017[M].

[10] Murray CJL, Lopez AD, Feehan DM, Peter ST, Yang G (2007). Validation of the symptom pattern method for analyzing verbal autopsy data. PLoS Med.

[11] Ferdous F, Ahmed S, Das SK, Chisti MJ, Nasrin D, Kotloff KL, Kotloff MM, Nataro JP, Ma E, Muhsen K, Yukiko Wagatsuma TA, Faruque AG (2018). Pneumonia mortality and healthcare utilization in young children in rural Bangladesh: a prospective verbal autopsy study. Trop Med Health.

[12] Hazard RH, Alam N, Chowdhury HR, Adair T, Alam S, Streatfield PK, Riley ID, Lopez AD (2018). Comparing tariff and medical assistant assigned causes of death from verbal autopsy interviews in Matlab, Bangladesh: implications for a health and demographic surveillance system. Popul Health Metrics.

[13] Karat AS, Maraba N, Tlali M, Charalambous S, Chihota VN, Churchyard GJ, Fielding KL, Yasmeen Hanifa Y, Suzanne Johnson S, McCarthy KM, Kathleen Kahn K, Daniel Chandramohan D, Grant AD (2018). Performance of verbal autopsy methods in estimating HIV-associated mortality among adults in South Africa. BMJ Glob Health.

[14] Omar A, Ganapathy SS, MFM A, Khoo YY, Jeevananthan C, Maria Awaluddin S, Yn JL, Rao C (2019). Cause-specific mortality estimates for Malaysia in 2013: results from a national sample verification study using medical record review and verbal autopsy. BMC Public Health.

[15] Polprasert W, Rao C, Adair T, Pattaraarchachai J, Porapakkham Y, Lopez AD (2010). Cause-of-death ascertainment for deaths that occur outside hospitals in Thailand: application of verbal autopsy methods. Popul Health Metrics.

[16] Thomas LM, D'Ambruoso L, Balabanova D (2018). Verbal autopsy in health policy and systems: a literature review. BMJ Glob Health.

[17] Tran HT, Nguyen HP, Walker SM, Hill PS, Rao C (2018). Validation of verbal autopsy methods using hospital medical records: a case study in Vietnam. BMC Med Res Methodol.

[18] Hazard RH, Buddhika MPK, Hart JD, Chowdhury HR, Firth S, Joshi R, Avelino F, Segarra A, Sarmiento DC, Azad AK, Ashrafi SAA, Bo KS, Kwa V, Lopez AD (2020). Automated verbal autopsy: from research to routine use in civil registration and vital statistics systems. BMC Med.

[19] World Health Organization (2007). Verbal autopsy standards: ascertaining and attributing cause of death.

[20] National data Annal by province. Beijing: National Bureau of Statistics of China; 2017. Available from: https://data.stats.gov.cn/english/easyquery.htm?cn=E0103 [cited 2019 March 14].

[21] Zhou M, Wang H, Zhu J, Chen W, Wang L, Liu S, Li YC, Wang LJ, Liu YN, Yin P, Liu JM, Yu SC, Tan F, RM MB, Coates MM, Dicker D, Fraser M, González-Medina D, Hamavid H, Hao YT, Hu GQ, Jiang GH, Kan HD, Lopez AD, Phillips MR, She J, Vos T, Wan X, Xu GL, Yan LL, Yu CH, Zhao Y, Zheng YF, Zou XN, Naghavi M, Wang Y, Murray CJL, Yang GH, Liang XF (2016). Cause-specific mortality for 240 causes in China during 1990-2013: a systematic subnational analysis for the Global Burden of Disease Study 2013. Lancet.

[22] Serina P, Riley I, Hernandez B, Flaxman AD, Praveen D, Tallo V, Joshi R, Sanvictores D, Stewart A, Mooney MD, Murray CJL, Lopez AD (2016). What is the optimal recall period for verbal autopsies? Validation study based on repeat interviews in three populations [J]. Popul Health Metrics.

[23] Nichols EK, Byass P, Chandramohan D, Clark SJ, Flaxman AD, Jakob R, Jakob R, Leitao J, Maire N, Rao C, Riley I, Setel PW (2018). The WHO 2016 verbal autopsy instrument: an international standard suitable for automated analysis by InterVA, InSilicoVA, and Tariff 2.0. PLoS Med.

[24] Murray CJ, Lozano R, Flaxman AD, Serina P, Phillips D, Stewart A, et al. Using verbal autopsy to measure causes of death: the comparative performance of existing methods. BMC Med. 2014;12(5). 10.1186/1741-7015-12-5. 10.1186/1741-7015-12-5 PMC3891983 24405531

[25] Institute for Health Metrics and Evaluation. Verbal Autopsy Tools-Tariff 2.0 Method [Internet]. Available from: http://www.healthdata.org/verbal-autopsy/tools [cited 2019 March 14].

[26] Serina P, Riley I, Stewart A, James SL, Flaxman AD, Lozano RS (2015). Improving performance of the Tariff Method for assigning causes of death to verbal autopsies. BMC Med.

[27] Feng J, Zhang L, Huang F, Yin JH, Tu H, Xia ZG, Zhou SS, Xiao N, Zhou XN (2018). Ready for malaria elimination: zero indigenous case reported in the People’s Republic of China. Malar J.

[28] GBD 2017 Causes of Death Collaborators (2018). Global, regional, and national age-sex-specific mortality for 282 causes of death in 195 countries and territories, 1980-2017: a systematic analysis for the Global Burden of Disease Study 2017. Lancet.

[29] Zhou M, Wang H, Zeng X, Yin P, Zhu J, Chen W, Li XH, Wang LJ, Wang LM, Liu YN, Liu JM, Zhang M, Qi JL, Yu SC, Afshin A, Gakidou E, Glenn S, Krish VS, Miller-Petrie MK, Mountjoy-Venning WC, Mullany EC, Redford SB, Liu HY, Naghavi M, Hay SI, Wang LH, Murray CJL, Liang XF (2019). Mortality, morbidity, and risk factors in China and its provinces, 1990-2017: a systematic analysis for the Global Burden of Disease Study 2017. Lancet (London, England).

[30] GBD 2017 Risk Factor Collaborators (2018). Global, regional, and national comparative risk assessment of 84 behavioural, environmental and occupational, and metabolic risks or clusters of risks for 195 countries and territories, 1990-2017: a systematic analysis for the Global Burden of Disease Study 2017. Lancet.

[31] Chen W, Xia C, Zheng R, Zhou M, Lin C, Zeng H, Zhang S, Wang LJ, Yang ZX, Sun KX, Li H, Brown MD, Islami F, Bray F, Jemal A, He J (2019). Disparities by province, age, and sex in site-specific cancer burden attributable to 23 potentially modifiable risk factors in China: a comparative risk assessment. Lancet Glob Health.

[32] Montazeri A (2004). Road-traffic-related mortality in Iran: a descriptive study. Public Health.

[33] Institute for Health Metrics and Evaluation. CRVS Resources and Tools Guidelines for interpreting verbal autopsy data. Available from: https://crvsgateway.info/file/11243/3231 [cited 2019 July 2].

[34] James SL, Flaxman AD, Murray CJ (2011). Performance of the Tariff Method: validation of a simple additive algorithm for analysis of verbal autopsies. Popul Health Metrics.

[35] Sankoh O, Byass P (2014). Time for civil registration with verbal autopsy. Lancet Glob Health.

[36] Gouda HN, Flaxman AD, Brolan CE, Joshi R, Riley ID, AbouZahr C (2017). New challenges for verbal autopsy: considering the ethical and social implications of verbal autopsy methods in routine health information systems. Soc Sci Med.

