# Supplementary material for: Estimating causes of out-of-hospital deaths in China: application of SmartVA methods
Source: Popul Health Metr. 2021 May 4;19:25. doi: 10.1186/s12963-021-00256-1 (PMC8097770; doi:10.1186/s12963-021-00256-1)
Supplement: Supplementary file 1 — Additional file 1: Appendix I. Gross Regional Product (GRP), urbanization rate, and life expectancy of the 9 pilot provinces. Appendix II. SmartVA Interview results from 9 pilot Provinces, 22 Districts between 2017 and 2018. Appendix III. The distribution of adult deaths from SmartVA and DSP, by sex ,age groups and urben-rural (%). Appendix IV. The distribution of adult deaths from determined and undetermined cause of death by sex and age groups (%). Appendix V. Percent of adult deaths due to the five leading causes of death and undetermined causes between SmartVA and GBD for China. [file 12963_2021_256_MOESM1_ESM.docx]

**Supplemental Materials for**

**Estimating causes of out-of-hospital deaths in China: application of SmartVA methods**

[Appendix 2](#_Toc64300202)

[Appendix Ⅰ Gross Regional Product (GRP), urbanization rate, and life expectancy of the 9 pilot provinces 2](#_Toc64300203)

[Appendix Ⅱ SmartVA Interview results from 9 pilot Provinces, 22 Districts between 2017 and 2018 3](#_Toc64300204)

[Appendix Ⅲ The distribution of adult deaths from SmartVA and DSP, by sex ,age groups and urben-rural (%) 4](#_Toc64300205)

[Appendix Ⅳ The distribution of adult deaths from determined and undetermined cause of death by sex and age groups (%) 5](#_Toc64300206)

[Appendix Ⅴ Percent of adult deaths due to the five leading causes of death and undetermined causes between SmartVA and GBD for China 6](#_Toc64300207)

# Appendix

## Appendix Ⅰ Gross Regional Product (GRP), urbanization rate, and life expectancy of the 9 pilot provinces

| Provinces | GRP (billion) | Urbanization rate (%) | Life expectancy | Region |
| --- | --- | --- | --- | --- |
| Anhui | 2701.8 | 53.5% | 74.5 | The Yangtze River Area |
| Guizhou | 1354.1 | 46.0% | 71.5 | Southwest Area |
| Heilongjiang | 1590.3 | 59.4% | 74.2 | Northeast Region |
| Henan | 7263.4 | 60.6% | 73.9 | Yellow River Area |
| Hubei | 3547.8 | 59.3% | 75.0 | The Yangtze River Area |
| Ningxia | 344.4 | 57.9% | 74.2 | The Big Northwest Area |
| Shaanxi | 2189.9 | 56.8% | 74.6 | Yellow River Area |
| Shandong | 4455.3 | 50.2% | 75.9 | North Coastal Area |
| Sichuan | 3698.0 | 50.8% | 73.7 | Southwest Area |

## Appendix Ⅱ SmartVA Interview results from 9 pilot Provinces, 22 Districts between 2017 and 2018

| Interview year | Province | Pilot Site | ≥12 years | | | |  | 29 days-11 years | | | |  | 0-28 days | | | |
| --- | --- | --- | --- | --- | --- | --- | --- | --- | --- | --- | --- | --- | --- | --- | --- | --- |
|  |  |  | N.O. of reported deaths | N.O. of interviews | Rejection (%) | Loss (%) |  | N.O. of reported deaths | N.O. of interviews | Rejection (%) | Loss (%) |  | N.O. of reported deaths | N.O. of interviews | Rejection  (%) | Loss (%) |
| 2017 | Shandong | Shanting | 446 | 420 | 0.2 | 5.6 |  | 7 | 7 | 0.0 | 0.0 |  | 3 | 3 | 0.0 | 0.0 |
|  | Henan | Yuanyang | 481 | 347 | 0.6 | 27.2 |  | 79 | 67 | 1.3 | 13.9 |  | 14 | 7 | 0.0 | 50.0 |
|  |  | Suixian | 439 | 347 | 2.7 | 18.2 |  | 87 | 65 | 10.3 | 14.9 |  | 4 | 3 | 0.0 | 25.0 |
|  | Hubei | Yidu | 414 | 380 | 0.5 | 7.7 |  | 20 | 19 | 5.0 | 0.0 |  | 7 | 7 | 0.0 | 0.0 |
|  |  | Yiling | 396 | 375 | 0.0 | 5.3 |  | 25 | 21 | 4.0 | 12.0 |  | 8 | 4 | 37.5 | 33.3 |
|  | Ningxia | Huinong | 647 | 392 | 1.4 | 38.0 |  | 14 | 9 | 7.1 | 28.6 |  | 3 | 1 | 33.3 | 100.0 |
|  |  | Zhongning | 446 | 362 | 0.9 | 17.9 |  | 8 | 7 | 0.0 | 12.5 |  | 0 | 0 | 0.0 | 0.0 |
|  |  | Helan | 510 | 373 | 4.1 | 22.8 |  | 33 | 22 | 18.2 | 15.2 |  | 10 | 5 | 40.0 | 10.0 |
|  | Shaanxi | Jingyang | 434 | 400 | 0.0 | 7.8 |  | 19 | 13 | 5.3 | 26.3 |  | 6 | 2 | 0.0 | 66.7 |
|  |  | Huayin | 401 | 388 | 3.2 | 0.0 |  | 13 | 11 | 15.4 | 0.0 |  | 4 | 4 | 0.0 | 0.0 |
|  |  | Meixian | 612 | 413 | 8.7 | 23.9 |  | 7 | 6 | 0.0 | 14.3 |  | 5 | 4 | 0.0 | 20.0 |
|  |  | Hanyin | 418 | 367 | 0.0 | 12.2 |  | 42 | 35 | 7.1 | 9.5 |  | 2 | 2 | 0.0 | 0.0 |
|  |  | Wangyi | 432 | 399 | 2.1 | 5.6 |  | 9 | 8 | 11.1 | 0.0 |  | 1 | 0 | 100.0 | 0.0 |
| 2018 | Heilongjiang | Suibin | 468 | 443 | 1.3 | 4.1 |  | 0 | 0 | 0.0 | 0.0 |  | 0 | 0 | 0.0 | 0.0 |
|  |  | Mishan | 905 | 846 | 0.7 | 5.9 |  | 0 | 0 | 0.0 | 0.0 |  | 0 | 0 | 0.0 | 0.0 |
|  |  | Luobei | 440 | 425 | 0.0 | 3.4 |  | 0 | 0 | 0.0 | 0.0 |  | 0 | 0 | 0.0 | 0.0 |
|  | Guizhou | Xishui | 857 | 812 | 0.0 | 5.3 |  | 8 | 6 | 25.0 | 0.0 |  | 0 | 0 | 0.0 | 0.0 |
|  |  | Kaiyang | 840 | 801 | 0.0 | 4.6 |  | 13 | 13 | 0.0 | 0.0 |  | 1 | 1 | 0.0 | 0.0 |
|  | Anhui | Huashan | 868 | 776 | 0.7 | 9.9 |  | 1 | 1 | 0.0 | 0.0 |  | 1 | 1 | 0.0 | 0.0 |
|  |  | Jingde | 835 | 832 | 0.0 | 0.4 |  | 5 | 5 | 0.0 | 0.0 |  | 0 | 0 | 0.0 | 0.0 |
|  | Sichuan | Pengan | 826 | 814 | 0.0 | 1.5 |  | 4 | 4 | 0.0 | 0.0 |  | 1 | 1 | 0.0 | 0.0 |
|  |  | Tongjiang | 1017 | 829 | 8.6 | 9.9 |  | 3 | 3 | 0.0 | 0.0 |  | 0 | 0 | 0.0 | 0.0 |
| Total |  |  | 13132 | 11541 | 232(1.8) | 1359(10.3) |  | 397 | 322 | 28(7.1) | 47(12.0) |  | 70 | 45 | 10(14.3) | 15(21.4) |

## Appendix Ⅲ The distribution of adult deaths from SmartVA and DSP, by sex ,age groups and urben-rural (%)

| Age Groups | Urben | | | | | |  | Urual | | | | | |
| --- | --- | --- | --- | --- | --- | --- | --- | --- | --- | --- | --- | --- | --- |
|  | Both | | Male | | Female | |  | Both | | Male | | Female | |
|  | Smartva | DSP | Smartva | DSP | Smartva | DSP |  | Smartva | DSP | Smartva | DSP | Smartva | DSP |
| 12-14 | 0.1 | 0.1 | 0.1 | 0.1 | 0.1 | 0.1 |  | 0.2 | 0.1 | 0.2 | 0.1 | 0.1 | 0.1 |
| 15-19 | 0.2 | 0.2 | 0.3 | 0.3 | 0.0 | 0.1 |  | 0.1 | 0.3 | 0.1 | 0.3 | 0.2 | 0.2 |
| 20-24 | 0.1 | 0.3 | 0.3 | 0.4 | 0.0 | 0.2 |  | 0.3 | 0.4 | 0.5 | 0.5 | 0.2 | 0.2 |
| 25-29 | 0.4 | 0.5 | 0.3 | 0.7 | 0.5 | 0.4 |  | 0.6 | 0.7 | 0.6 | 0.8 | 0.5 | 0.4 |
| 30-34 | 0.3 | 0.7 | 0.4 | 0.8 | 0.2 | 0.5 |  | 0.4 | 0.8 | 0.6 | 1.0 | 0.3 | 0.5 |
| 35-39 | 0.7 | 0.9 | 0.7 | 1.1 | 0.7 | 0.7 |  | 0.8 | 1.1 | 0.8 | 1.4 | 0.7 | 0.7 |
| 40-44 | 0.7 | 1.7 | 0.8 | 2.1 | 0.6 | 1.2 |  | 1.6 | 1.9 | 1.9 | 2.4 | 1.2 | 1.3 |
| 45-49 | 2.2 | 3.1 | 2.7 | 3.7 | 1.7 | 2.2 |  | 2.9 | 3.5 | 3.6 | 4.3 | 2.1 | 2.5 |
| 50-54 | 3.6 | 4.7 | 4.7 | 5.7 | 2.2 | 3.2 |  | 4.4 | 5.2 | 5.5 | 6.1 | 3.1 | 3.8 |
| 55-59 | 2.7 | 4.8 | 3.6 | 6.0 | 1.6 | 3.3 |  | 4.6 | 4.7 | 5.8 | 5.6 | 3.1 | 3.5 |
| 60-64 | 7.3 | 8.1 | 9.4 | 9.7 | 4.9 | 6.0 |  | 8.1 | 8.5 | 9.1 | 9.8 | 7.0 | 6.7 |
| 65-69 | 8.6 | 9.4 | 10.0 | 10.6 | 7.0 | 7.6 |  | 10.9 | 10.4 | 12.2 | 11.6 | 9.4 | 8.8 |
| 70-74 | 9.6 | 10.6 | 10.8 | 11.4 | 8.2 | 9.6 |  | 13.7 | 11.7 | 14.0 | 12.4 | 13.3 | 10.8 |
| 75-79 | 16.2 | 14.0 | 17.6 | 13.9 | 14.6 | 14.2 |  | 16.4 | 14.4 | 15.9 | 14.4 | 17.0 | 14.3 |
| 80-84 | 21.7 | 17.4 | 19.6 | 15.7 | 24.0 | 19.8 |  | 16.8 | 16.3 | 15.2 | 14.7 | 18.9 | 18.7 |
| 85+ | 25.6 | 23.3 | 18.8 | 17.8 | 33.7 | 30.9 |  | 18.1 | 20.1 | 14.1 | 14.7 | 23.1 | 27.6 |
| Total | 2,782 | 728,975 | 1,507 | 421,719 | 1,275 | 307,256 |  | 8,738 | 1,278,365 | 4,852 | 743,617 | 3,886 | 534,748 |
|  | (100.0) | (100.0) | (100.0) | (100.0) | (100.0) | (100.0) |  | (100.0) | (100.0) | (100.0) | (100.0) | (100.0) | (100.0) |

## Appendix Ⅳ The distribution of adult deaths from determined and undetermined cause of death by sex and age groups (%)

| Age group | Both | | | Male | | | Female | | |
| --- | --- | --- | --- | --- | --- | --- | --- | --- | --- |
|  | SmartVA | Determined cause of death | Undetermined cause of death | SmartVA | Determined cause of death | Undetermined cause of death | SmartVA | Determined cause of death | Undetermined cause of death |
| 12-14 | 0.1 | 0.1 | 0.2 | 0.2 | 0.2 | 0.2 | 0.1 | 0.1 | 0.2 |
| 15-19 | 0.1 | 0.1 | 0.4 | 0.2 | 0.1 | 0.4 | 0.1 | 0.1 | 0.5 |
| 20-24 | 0.3 | 0.3 | 0.1 | 0.4 | 0.4 | 0.2 | 0.1 | 0.1 | 0.0. |
| 25-29 | 0.5 | 0.5 | 0.7 | 0.6 | 0.5 | 0.9 | 0.5 | 0.5 | 0.5 |
| 30-34 | 0.4 | 0.4 | 0.3 | 0.6 | 0.5 | 0.7 | 0.3 | 0.3 | 0.0 |
| 35-39 | 0.7 | 0.7 | 1.0 | 0.8 | 0.7 | 1.1 | 0.7 | 0.7 | 0.8 |
| 40-44 | 1.4 | 1.4 | 1.5 | 1.7 | 1.6 | 1.9 | 1.0 | 1.0 | 1.1 |
| 45-49 | 2.7 | 2.7 | 2.9 | 3.3 | 3.3 | 4.1 | 2.0 | 2.0 | 1.8 |
| 50-54 | 4.2 | 4.3 | 2.9 | 5.3 | 5.4 | 3.9 | 2.9 | 3.0 | 20 |
| 55-59 | 4.1 | 4.3 | 2.9 | 5.3 | 5.4 | 3.7 | 2.8 | 2.8 | 2.1 |
| 60-64 | 8.0 | 8.1 | 6.3 | 9.2 | 9.4 | 6.6 | 6.5 | 6.5 | 6.0 |
| 65-69 | 10.4 | 10.7 | 7.3 | 11.7 | 12 | 8.1 | 8.8 | 9.1 | 6.7 |
| 70-74 | 12.7 | 13.1 | 9.3 | 13.3 | 13.7 | 8.8 | 12.1 | 12.4 | 9.8 |
| 75-79 | 16.3 | 16.7 | 13.4 | 16.3 | 16.4 | 15.4 | 16.4 | 17.0 | 11.7 |
| 80-84 | 18.0 | 18.0 | 18.5 | 16.2 | 16.1 | 17.6 | 20.2 | 20.3 | 19.2 |
| 85+ | 19.9 | 18.5 | 32.3 | 15.2 | 14.2 | 26.4 | 25.7 | 24.1 | 37.5 |
| total | 11,520(100.0) | 10373(100.0) | 1147(100.0) | 6,359(100.0) | 5825(100.0) | 534(100.0) | 5,161(100.0) | 4548(100.0) | 613(100.0) |

## Appendix Ⅴ Percent of adult deaths due to the five leading causes of death and undetermined causes between SmartVA and GBD for China

| Gender | Ranking | 15-49 | | | 50-69 | | | ≥70 | | |
| --- | --- | --- | --- | --- | --- | --- | --- | --- | --- | --- |
|  |  | CoD | % of total | GBD for China | CoD | % of total | GBD for China | CoD | % of total | GBD for China |
| Male | 1 | Ischemic Heart Disease | 8.9  (6.3~11.5) | 10.7  (10.3~11) | Stroke | 18.8  (17.1~20.5) | 19.2  (18.8~19.6) | Stroke | 23.7  (22.3~25) | 23.0  (22.6~23.6) |
|  | 2 | Stroke | 8.9  (6.3~11.5) | 10.6  (10.2~10.9) | Ischemic Heart Disease | 15.5  (13.9~17.1) | 14  (13.5~14.3) | Ischemic Heart Disease | 20.2  (19~21.5) | 18.2  (17.8~18.7) |
|  | 3 | Cirrhosis | 7.8  (5.4~10.3) | 3.9  (3.6~4.7) | Lung Cancer | 14.8  (13.3~16.4) | 11.4  (10.9~11.8) | Chronic Respiratory | 13.8  (12.7~14.9) | 14.1  (13.8~14.4) |
|  | 4 | Falls | 6.6  (4.3~8.8) | 3.2  (2.1~3.6) | Chronic Respiratory | 6.6  (5.5~7.7) | 5.7  (5.5~6.0) | Prostate Cancer | 5.1(4.4~5.7) | 1.2  (1~1.6) |
|  | 5 | Road Traffic | 6.4  (4.2~8.6) | 15.5  (14.8~15.9) | Prostate Cancer | 5.8  (4.7~6.8) | 0.5  (0.5~0.7) | Lung Cancer | 4.5  (3.8~5.1) | 6.7  (6.4~6.9) |
|  | Undetermined | Undetermined | 10.6  (7.8~13.4) | 0 | Undetermined | 6  (4.9~7) | 0 | Undetermined | 9.4  (8.5~10.3) | 0 |
|  | Total | total | 472 |  | total | 1997 |  | total | 3877 |  |
| Female | 1 | Cervical Cancer | 8.6  (5.1~12.1) | 3.7  (2.3~4.1) | Stroke | 21.5  (19.1~24) | 19.8  (19.3~20.4) | Stroke | 28  (26.6~29.5) | 22.4  (21.8~22.9) |
|  | 2 | Ischemic Heart Disease | 8.2  (4.8~11.6) | 7.9  (7.6~8.4) | Ischemic Heart Disease | 20.3  (17.9~22.7) | 13.7  (13.2~14.1) | Ischemic Heart Disease | 24.7  (23.3~26) | 21.5  (20.7~22) |
|  | 3 | Leukemia/Lymphomas | 7.8  (4.4~11.1) | 2.6  (2~3) | Leukemia/Lymphomas | 5.6  (4.2~6.9) | 0.8  (0.6~0.9) | Chronic Respiratory | 10.4  (9.5~11.4) | 12.3  (11.5~13.1) |
|  | 4 | Stroke | 7.8  (4.4~11.1) | 9.6  (9.2~10.2) | Cervical Cancer | 5.2  (3.9~6.5) | 2.2  (1.4~2.3) | Diabetes | 3  (2.5~3.6) | 1.6  (1.6~1.7) |
|  | 5 | Breast Cancer | 7.4  (4.1~10.7) | 6  (5.5~6.8) | Diabetes | 4.2  (3~5.4) | 2.6  (2.5~2.7) | Pneumonia | 2.3  (1.8~2.8) | 2  (1.8~2.6) |
|  | Undetermined | Undetermined | 11.9  (7.8~15.9) | 0 | Undetermined | 9.6  (7.8~11.3) | 0 | Undetermined | 12.5  (11.5~13.6) | 0 |
|  | Total | total | 244 |  | total | 1077 |  | total | 3838 |  |
